# Supplementary material for: Topological soliton molecule in quasi 1D charge density wave
Source: Nat Commun. 2023 Aug 22;14:5085. doi: 10.1038/s41467-023-40834-5 (PMC10444770; doi:10.1038/s41467-023-40834-5)
Supplement: Supplementary file 1 — Supplementary Information [file 41467_2023_40834_MOESM1_ESM.pdf]

Supplementary information for Topological soliton molecule in quasi 1D charge density wave

Taehwan Im, Sun Kyu Song, Jae Whan Park, Han Woong Yeom

August 11, 2023

Z_4 charge density wave state and chiral solitons on the In/Si(111) surface

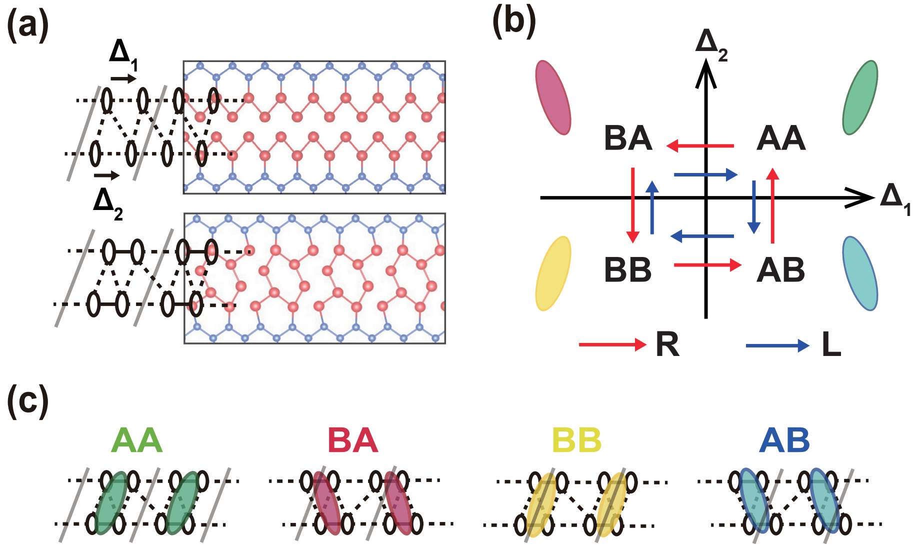

Fig. S1: (a) Atomic structures of the normal (upper pannel) and charge-density-wave (bottom pannel) states of the In/Si(111) surface. Blue and red balls represent Si and In atoms on the topmost layer. These structures can be mapped into a simpler model structure of two chains of atoms (the double Su-Schrieffer-Higger chain model) with equivalent electronic band structures as shown in the left. Both intra- and inter-chain hoppings are active between the two chains. The dimerized bonds are depicted by thick solid lines for the CDW state. The CDW state is thus characterized by the dimerization on the upper and bottom chains, which is represented by two order parameters, Δ_1 and Δ_2, respectively. (b) and (c) The CDW state are four fold degenerated by two dimerizations on upper or bottom chain (left and right or $\pm\Delta_1$ or $\pm\Delta_2$), as depicted by CDW maxima of different color and denoted by AA, BA, BB, and AB. These different CDW states coexist as connected by soliton domain walls. The relationship between different CDW states and the right-chiral (RCS) and left-chiral (LCS) solitons is indicated in (b). Not shown here, but the third type of a soliton, a non-chiral soliton connects between AA and BB or BA and AB CDW states (diagonally in (b)).

The electronic states of right- and left-chiral solitons

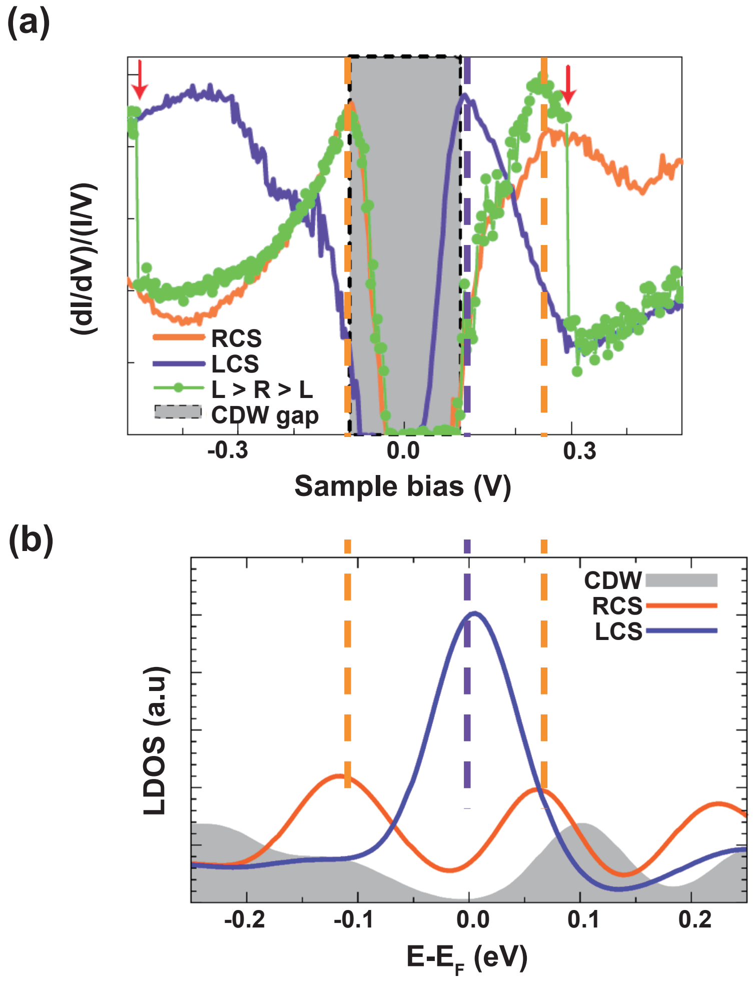

Fig. S2: (a) The STS spectra for the right and left chiral solitons (adapted from *Kim, T.H., Cheon, S. & Yeom, H.W. Nature Phys.* ***13****, 444–447 (2017)*, shown in orange and blue lines, respectively. (b) Our DFT calculation for the LDOS of the right and left chiral solitons given in the same color. The grey region corresponds to the LDOS of the pristine In wire. The in-gap states of the experiment and calculation agree well when we rescale the energy of the DFT calculations to match the band gap size of the CDW state, as mentioned in the main text. That is, the present DFT calculations largely underestimate the CDW band gap.

Filled and empty state STM images for a collection of defects and solitons

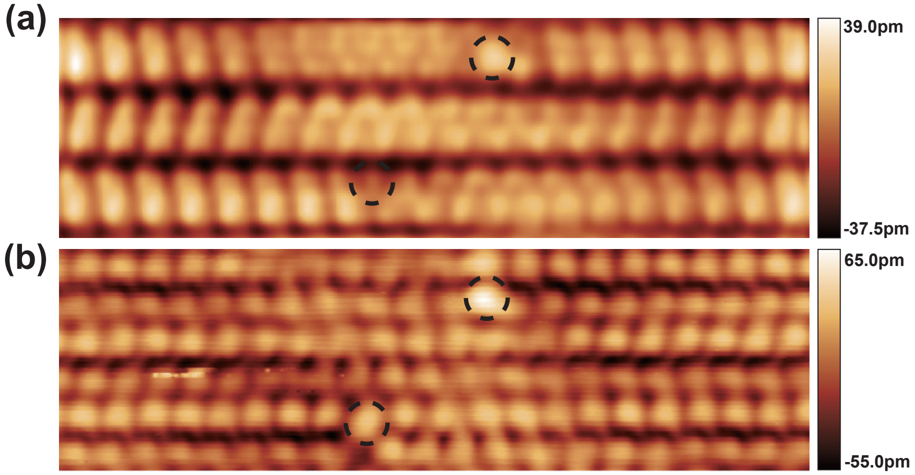

Fig. S3: (*a*) Filled- and (*b*) empty-state STM images ($V_t = \pm 500mV$) for a part of a 2D domain wall with solitons and defects as shown in Fig. 1. Defects are indicated by dashed circles, which appear as strong bright protrusions in empty states.

Structural influence of the defects and solitons in the neighboring chains

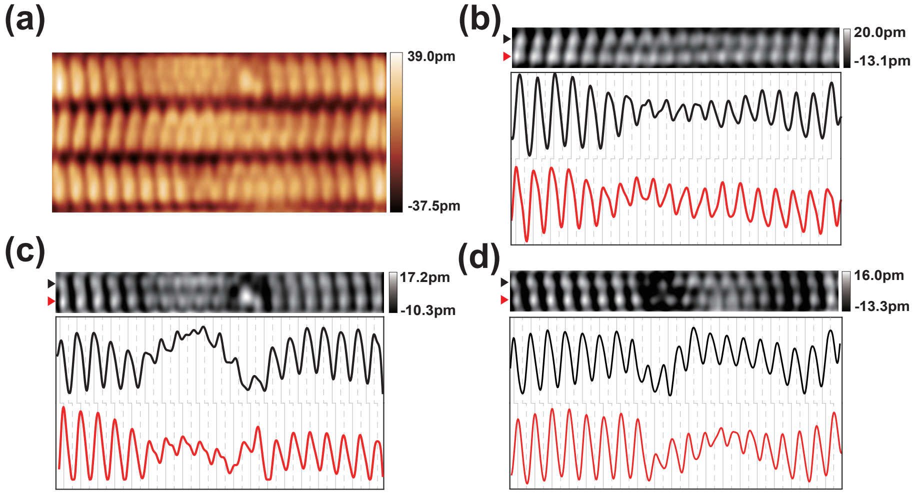

Fig. S4: (a) The STM image of three In chains shown in Fig. 1 with the central wire featuring a soliton molecule. (b) The enlarge image of the central wire with its line profiles for the upper and bottom chains as shown in Fig. 1. (c) The same image and line profile for the top wire with a soliton and a defect. (d) The same images and line profiles for the bottom wire, which also has a soliton and a defect. Each image and line profile are removed with slowly varying backgrounds and high-pass-filtered. One can assure that the strong local distortions of the defect do not propagate to the central wire. The same conclusion can be obtained by the STM images and DFT calculations for an isolated defect surrounded by pristine wires. The lattice distortion induced by a defect or a soliton in the neighboring wire is marginal. The high-pass filtering was carried out by cutting out the fluctuating signals with an amplitude below 10 pm.

Comparison of STM images and line profiles at two different bias voltages

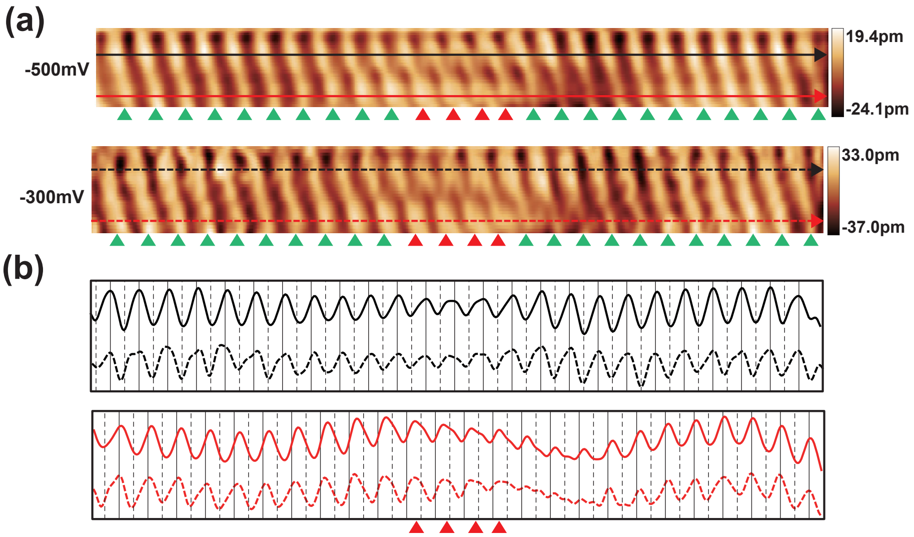

Fig. S5: (a) The STM images at the bias of -500 and -300 mV. (b) Comparison of line profiles of top (black lines) and bottom (red lines) chains measured at two different biases. The solid black and red lines (dashed lines) represent the line profiles measured along the arrows of the solid lines (dashed lines) shown in (a), respectively. The CDW patterns are inverted 180 degrees compared to the Fig 1 in manuscript (corresponding to a neighboring wire in the 8×2 CDW unit), so the soliton also has an inverted shape with their phase shifts in the bottom chains.

STS line profile showing the phase shift in the electronic states

(a)

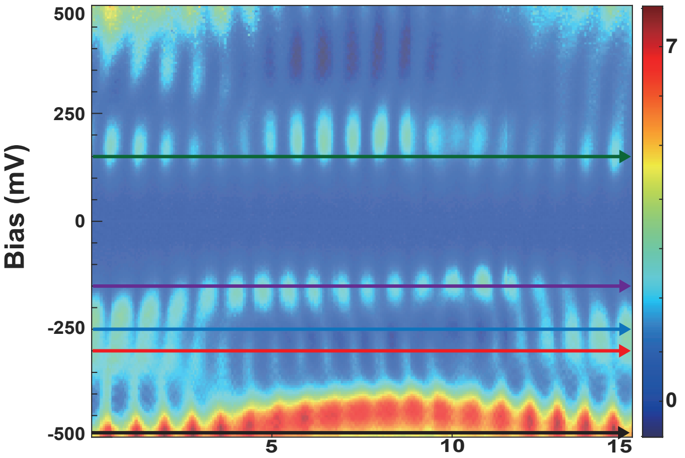

(b)

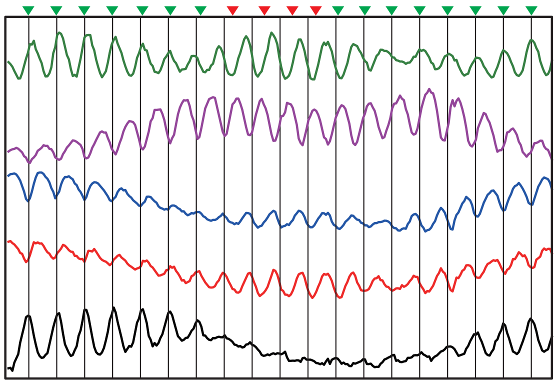

Fig. S6: (a) The STS (normalized dI/dV) map shown in Fig. 2 and (b) its line plots at specified energies shown as colored arrows in (a). These line plots corresponds to the local modulation of electronic states at specific energies and those near the band bap (at +- 0.2-0.3 eV, green, purple, blue, and red lines) are important for the CDW states. All these electronic states show the CDW modulations (out of phase between filled and empty states). Crossing the soliton molecule, all the modulation shows the phase shift, which but result in a net zero total phase shift. The exact location of the phase shifts along the chains (along the longitudinal direction of the soliton molecule) is slightly different between the filled and the empty states, but is consistent with the locations of the soliton molecular states in filled and empty states. Green and red triangles above the line profile graph represent the positions of in-phase and out-of-phase CDW maxima in topography image as shown Fig 1f and S5.

STM images and STS spectra for four different cases of soliton molecules

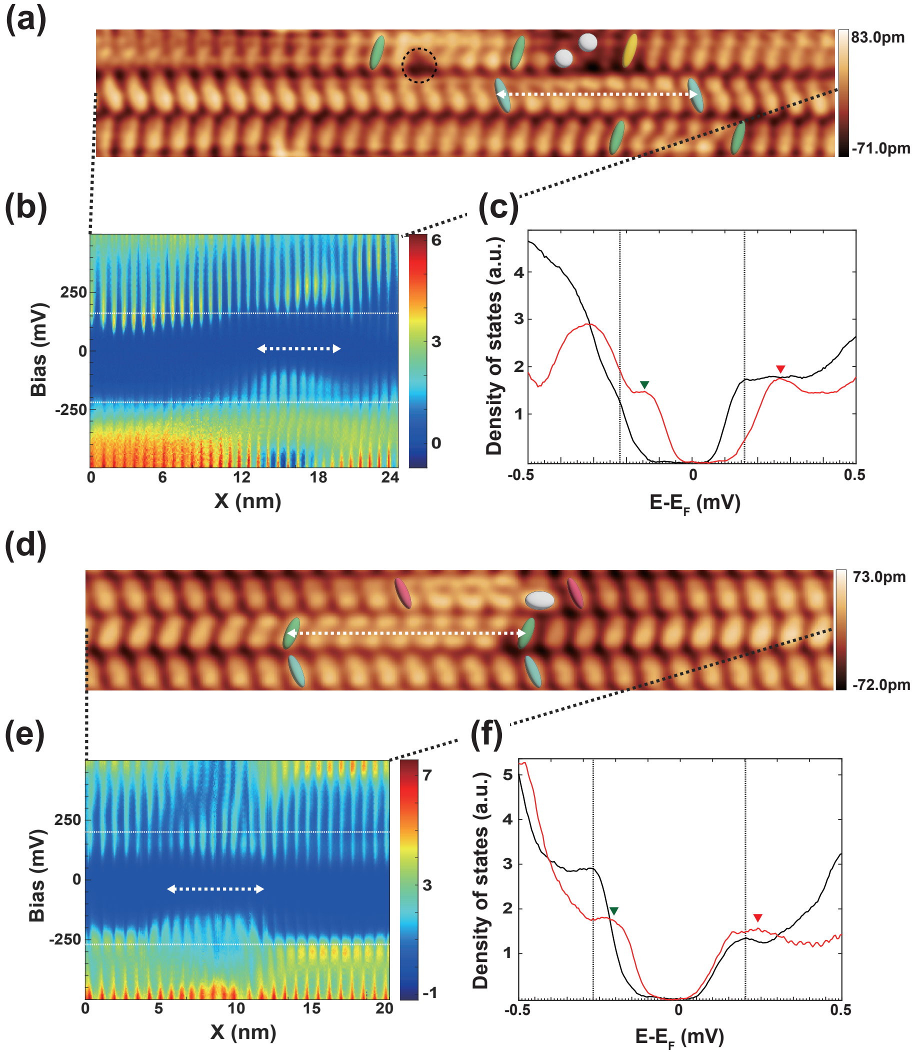

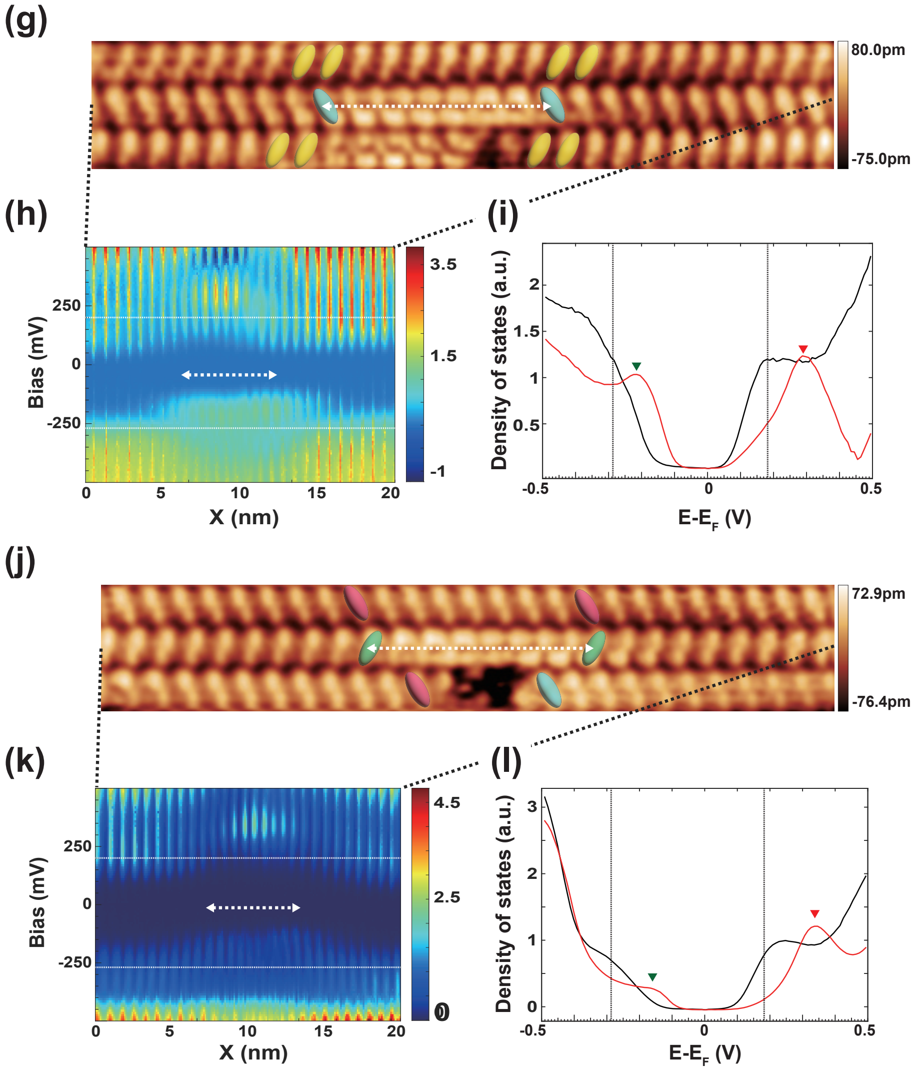

Fig. S7: (a), (d), (g) and (j) STM images for clusters of defects and solitons, which include single soliton molecule in the central wire. The positions of defects and solitons in the top and bottom wires are specified. (b), (e), (h) and (k) Corresponding STS line scan data of the central wires crossing the soliton molecule. (c),(f), (i) and (l) Averaged point STS spectra for a pristine CDW unitcell (black) and a soliton molecule (red) in (a), (d), (g) and (j) respectively. Vertical doted lines represent the edge of the CDW energy gap, which is also specified in (c), (f), (i) and (l) with horizontal lines. The in-gap molecular state above the valence band edge is very clear for all cases together with the enhanced density of states near the conduction band edge. Every STS data is a measurement of the center of the central wire.

Details of the high-pass filtering method

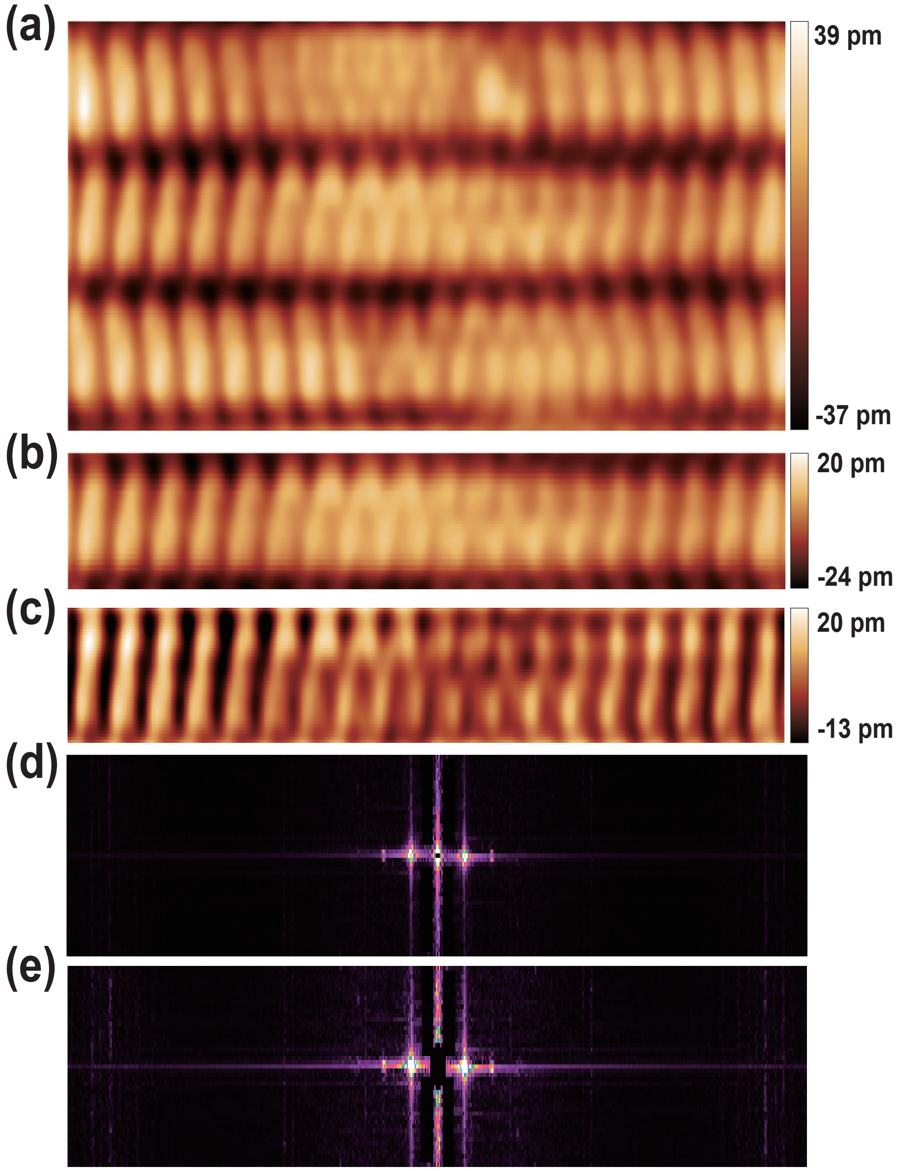

Fig. S8: (a) Original filled state STM image at $V_t = -500mV$ as shown in Fig. 1 of the main text. (b) An image of the central wire that is flattened by removing a slowly varying background and (c) its high-pass filtered image with cutting-off a Γ-point peak from its 2D FFT [(d) the FFT image and (e) that after the cut-off] and offset of 3 pm.

Phase shift map along a soliton molecule

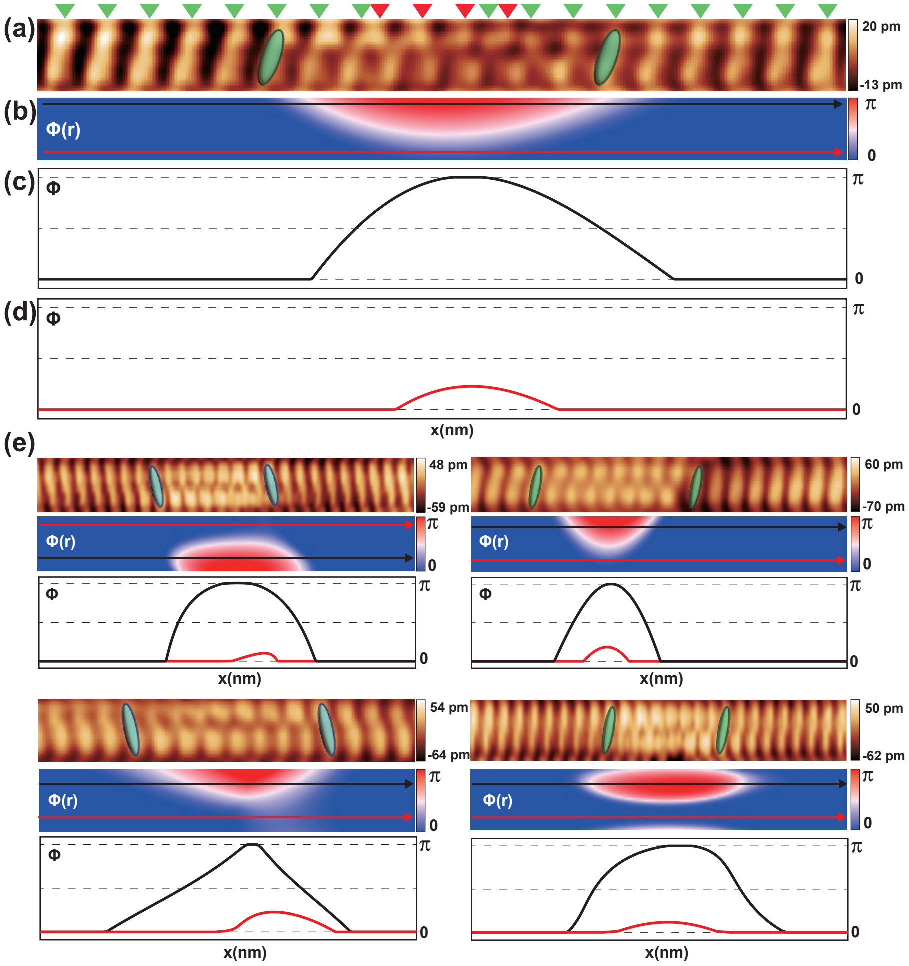

Fig. S9: (*a*) The filled state STM image and (*b*) its phase map which is obtained by the "Lock-in" technique [(a) Filled-state STM image of a soliton molecule (shown in Fig. 1 of the manuscript) and (b) its phase map as obtained by the lock-in technique [J.A. Slezak et al., PNAS 105(9) 3203-3208(2008); A. Mesaros, et al. Science 333, 426(2011)]. Line cuts of the phase map along the (c) upper and (d) lower In chains shown with matched colors in (b). The double phase shift exists only one In chains manifesting the existence of two chiral solitons in close proximity. (e) The same sets of data for four other soliton molecule cases given in Fig. S7.

3D STM topographic image of a soliton molecule

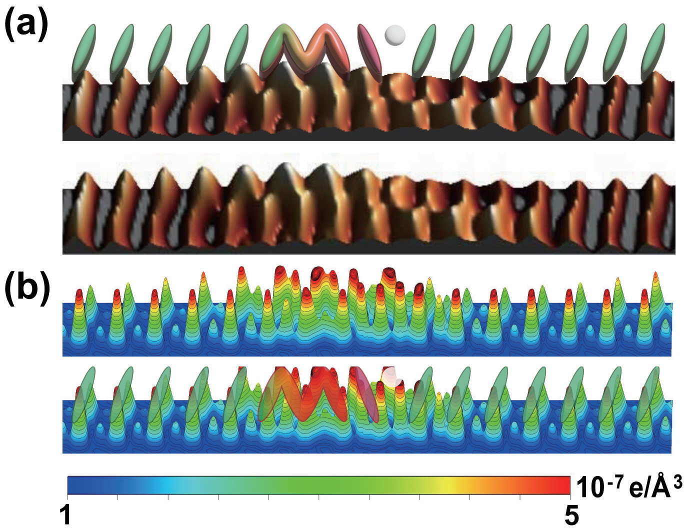

Fig. S10: (a) 3D rendering of the soliton molecule part of the STM image shown in Fig. 1. The top and bottom images are the same but the schematic CDW and soliton shapes are added on the top image. (b) 3D rendering of the simulated STM image (the charge density plot integrated over the energy range of -0.2 to 0 eV). The contour interval is set to $3 \times 10^{-8} e/\AA^3$. The top and bottom are the same images but the schematic CDW and soliton shapes are added on the bottom image.

Comparison of soliton included in soliton molecule and isolated soliton.

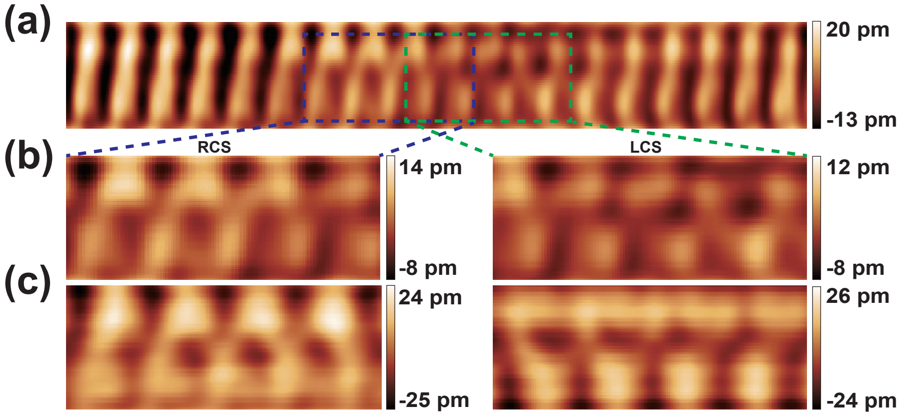

Fig. S11: (a) A high-pass filtered STM image shown in Fig. 1 and (b) its enlarged images of the right- and left-chiral soliton regions. The blue (green) dotted square region in (a) represents the right (left) chiral soliton. (c) Enlarged images of the isolated right- and left-chiral solitons shown in Fig. 1.

Relaxation of atomic structures (for Supplementary Fig. S12 - 14)
Supplementary Fig. 12 presents the soliton-molecule energy as a function of the soliton-soliton distance. Initially, we relaxed all indium atoms except the eight indium atoms situated between the two solitons, which are indicated by ovals in Supplementary Fig. 12(a). These atoms were fixed during the first stage of the relaxation process. Subsequently, we fully relaxed all indium atoms from the final structure of the first stage, including the eight indium atoms. The term "fixed" denotes that these atoms were fixed during the initial stage of the relaxation process. When we relax all atoms from the beginning, the two solitons merge and annihilate each other into the pristine structure. For instance, for the $5a_0$-distance, the initial structure is directly converged to the ground state by full-relaxation without any constraint, as shown in Supplementary Fig. 13(a). We further verified the full-relaxed structure from the Supplementary Fig. 13(b) structure to obtain the true local minimum structure, as demonstrated in Supplementary Fig. 13(c). However, during full relaxation, the soliton can move, making it difficult to control the soliton-soliton distance, as observed in Supplementary Fig. 13(e) and 13(f). Therefore, we have plotted the energetics for both cases in Supplementary Fig. 12(b), and all partially and fully relaxed structures are provided in Supplementary Fig. 14.

DFT calculations for the energetics of a soliton molecule

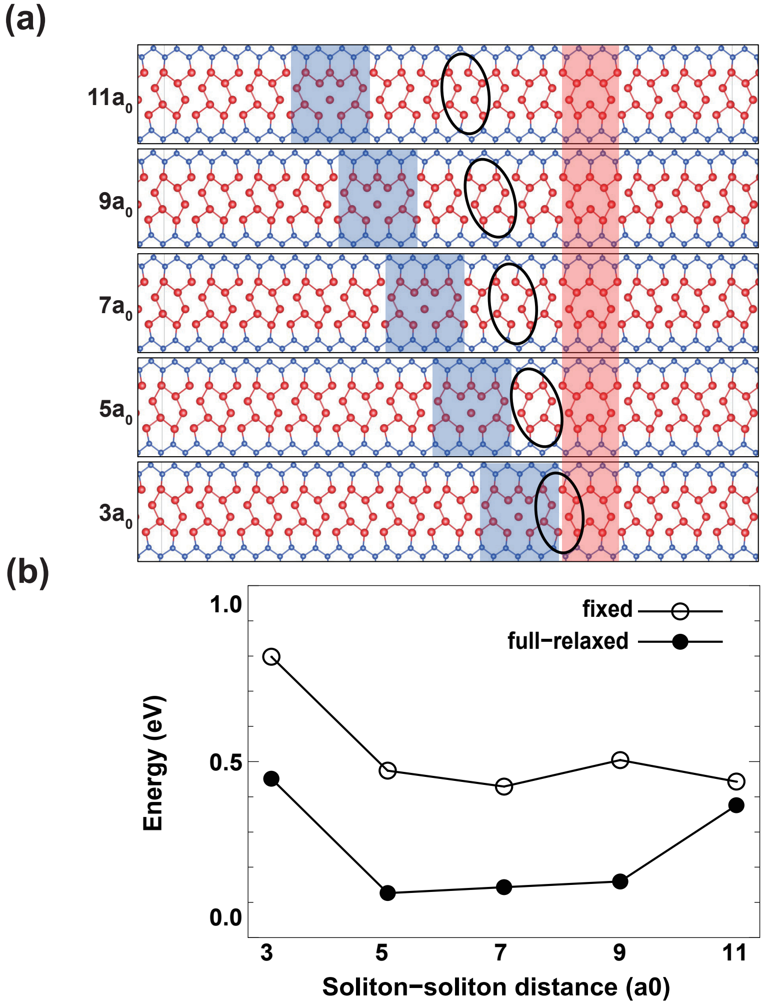

Fig. S12: We calculate the total energy of two wires (a 8×24 supercell), where one contains two solitons (right- and left-chiral solitons) with a different separation as shown in (a). The eight In atoms in the middle of the two solitons are fixed or relaxed and their formation energy variations are all plotted in (b). The formation energy is defined by $E_{formation} = E_{RCS+LCS}$-$E_{CDW}$, where $E_{RCS+LCS}$ and E_{CDW} are the total energy of the supercell with two solitons and without soliton (pristine CDW structure), respectively.

Atomic structure of soliton molecules

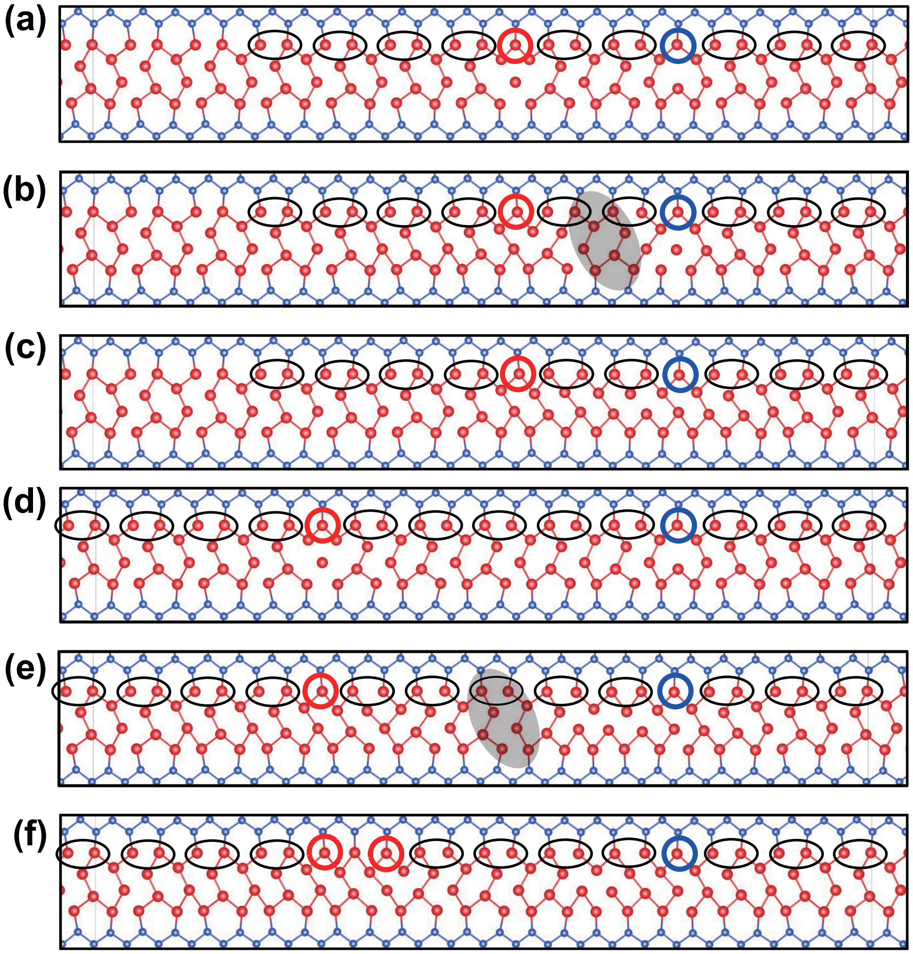

Fig. S13: (a)-(b) $5a_0$ distance. (d)-(f) $11a_0$ distance. (a), (d) Initial structure. (b), (e) The eight In atoms in the middle of the two solitons are fixed. (c), (f) Full-relaxed from the (b) and (e) structure, respectively. Black ellipses include fixed eight In atoms. Blue(Red) circles represent right(left) chiral solitons.

Atomic structure as a function of soliton-soliton distance ($3a_0 \sim 11a_0$).

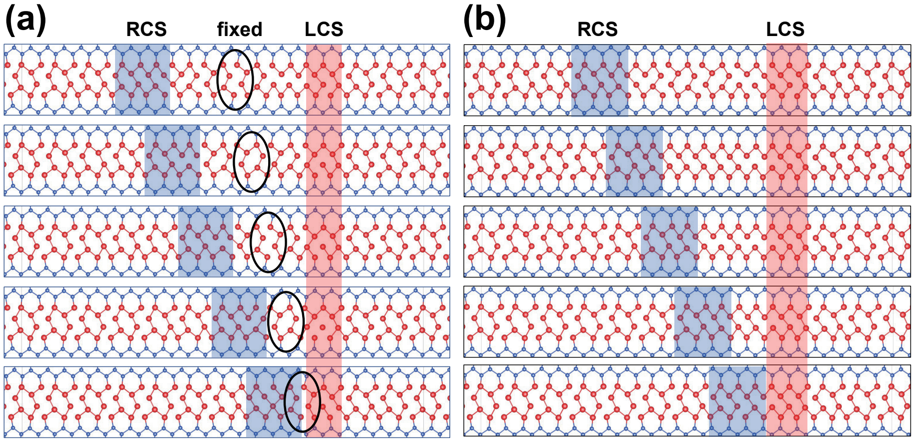

Fig. S14: (a) Full relaxed structure except the eight In atoms in the middle of two solitons (solid ovals). (b) Full relaxed structure including the eight In atoms. For (b), we used the final structure in (a) as the initial stage.

Comparison of a typical dI/dV and I-V curve

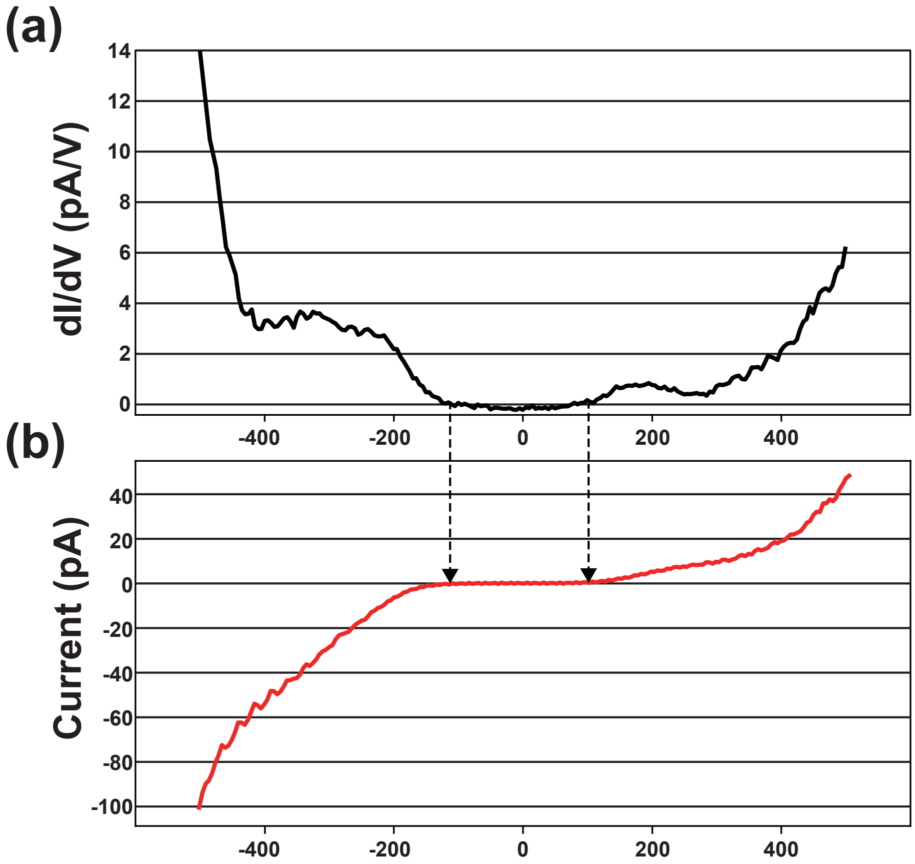

Fig. S15: (a) dI/dV and (b) I-V curve obtained by a point STS measurement at 2.3 nm locations on a central wire containing a soliton molecule shown in Fig. 1 of the main text.

Band-gap size (Supplementary Fig. S16 - 18)

The LDA calculations predict a clear insulating character for the present system, as seen in the band structure and LDOS in Supplementary Fig. S17. However, the predicted band gap of 0.1 eV is much smaller than the experimental value of 0.4 eV. This issue has been previously addressed and can be partly improved using more sophisticated functionals such as hybrid functional [PRB 102, 121408(R) (2020)] or more accurately by performing GW quasiparticle calculations [PRB 99, 155107 (2019)]. It's important to note that the underestimation of the band gap does not affect the prediction of the ground state structure and band dispersions by LDA calculations. As shown in Supplementary Fig. S18 with the comparison of the hybrid functional calculation, the current LDA calculation, and the GW calculation, the band dispersions are large consistent between different calculations but the LDA-based DFT simply underestimate the band gap size. For a better comparison, one can simply put an extra energy gap in LDA [see Supplementary Fig. S18(c) and S18(d)]. This correction corresponds to considering an extra momentum-independent self-energy, which is not reflected in the LDA calculation [PRB 85, 195111 (2012)]. Note that the red circled part around Γ point in the hybrid functional calculation is an artifact of this calculation, which deviates from the experimental results and the GW calculation. There still remains differences between the LDA and GW calculations such as the energy of the Si bulk valence band top and the band width of the valence bands of the In wires. The latter is not relevant with the present discussion of In wires. Note also that the topological property of the present system is decided by the band structure around the CDW gap at the X point of the Brillouin zone. For this part, the GW, hybrid-functional, and the band-gap-corrected LDA calculations agree very well.

The theoretical LDOS was plotted with an inserted gap of 0.18 eV between filled and empty states (Supplementary Fig. S16). This figure also compares the different Gaussian broadening of the calculated LDOS of $\sigma = 0.01$ and 0.04 eV ((a) and (b), respetively). This broadening explains the thermal and instrumentational broadening of the experiment. The shape of the theoretical LDOS at the small smearing value is more complicated compared to the STS spectra (Fig. S17).

Comparison between experimental STS spectra (left panel) and theoretical LDOS (right panel)

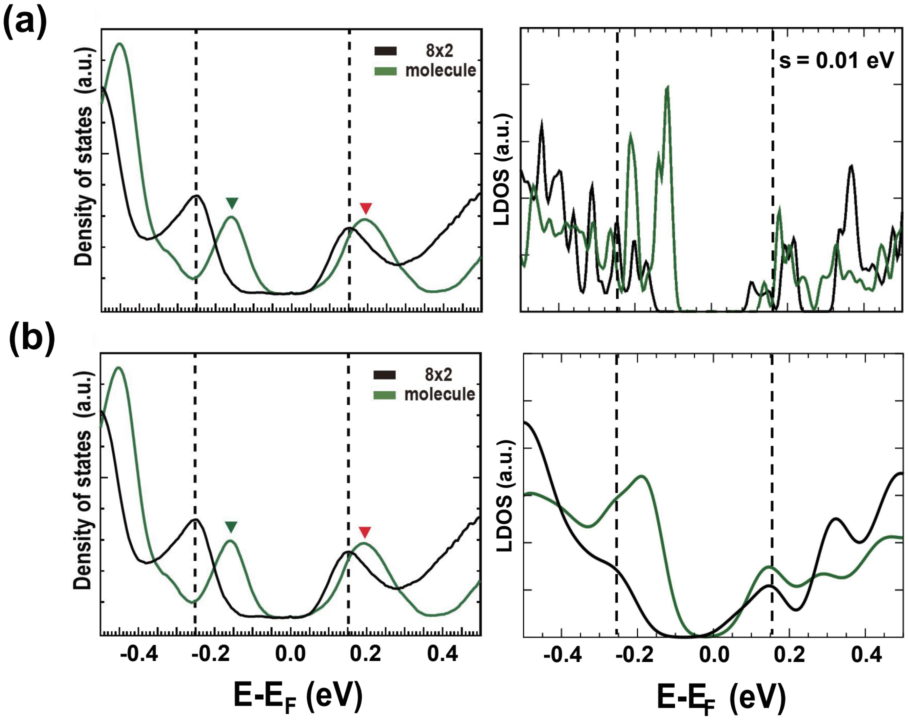

Fig. S16: (a) Calculated LDOS with a gap of 0.18 eV inserted and an energy broadening of $\sigma = 0.01$ eV. (b) The same calculated LDOS with a extra broadening of 0.04 eV from that of (a).

Theoretical structure and LDOS of a pristine CDW (8x2) state.

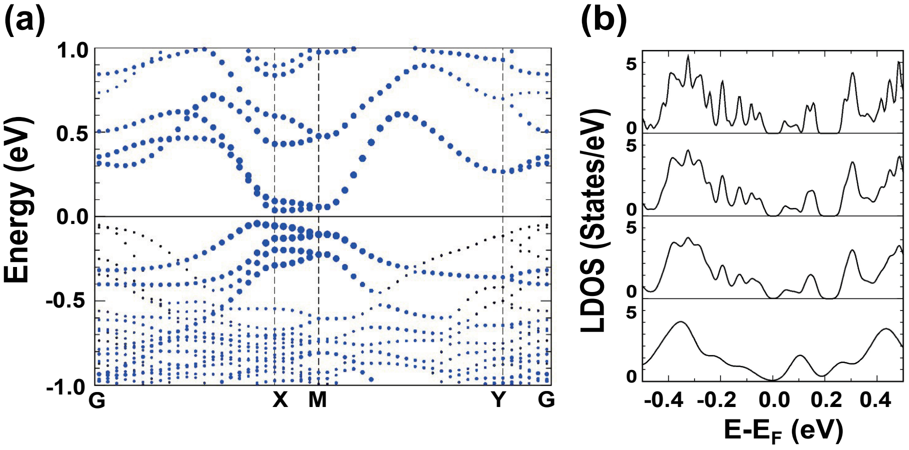

Fig. S17: (a) Band dispersions of the CDW ground state in the present LDA-based DFT calculation. (b) LDOS for the ground state as a function of the Gaussian smearing (σ) of 0.01, 0.15, 0.02, and 0.05 eV from the top.

Comparison of theoretical band dispersions obtained by different calculation schemes

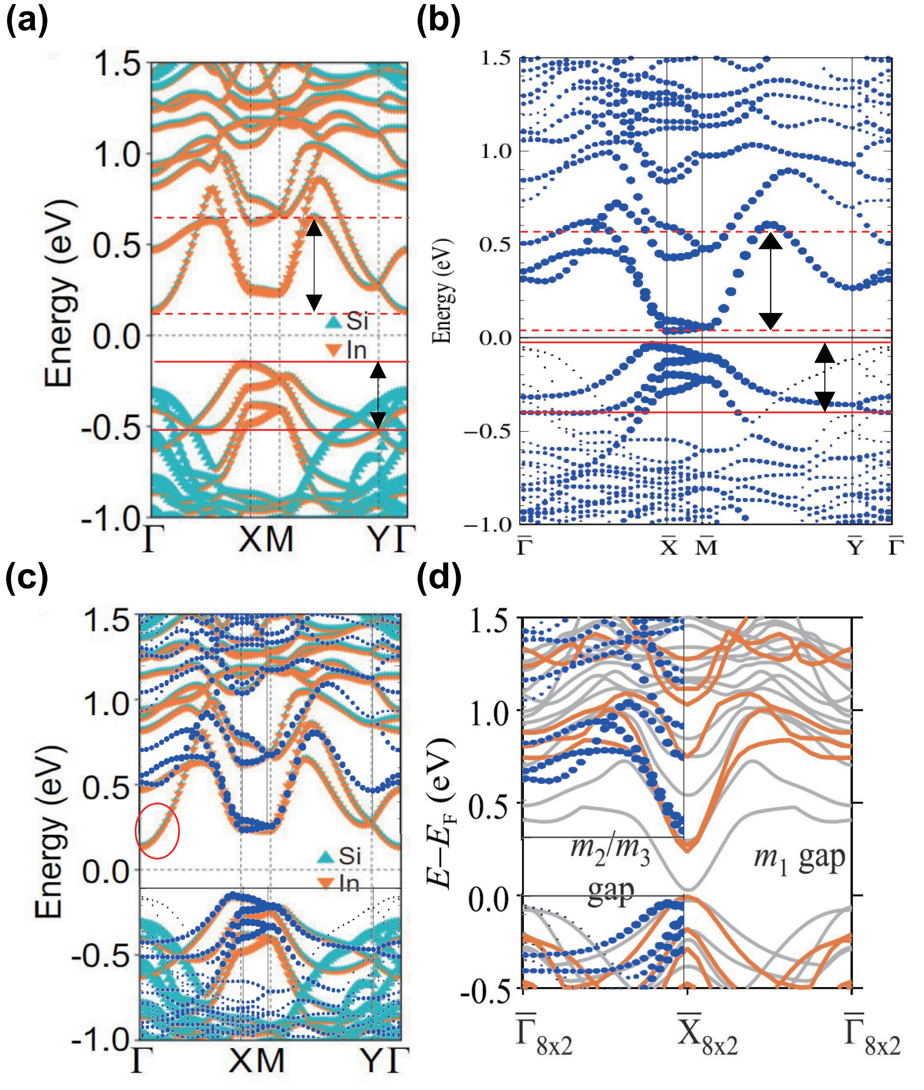

Fig. S18: (a) Calculated results using Heyd-Scuseria-Ernzerhof (HSE06) hybrid functionals [Phys. Rev. B 102, 121408(R) (2020)]. (b) Present LDA calculation. (c) A comparison of HSE (a) and LDA (b) overlaid. (d) Resuls of single-particle Green's function calculation with screened Coulomb interaction (so called the GW calculation) [Phys. Rev. B 99, 155107 (2019)].
